# Individual and residency program factors related to depression, anxiety and burnout in physician residents – a Brazilian survey

**DOI:** 10.1186/s12888-022-03916-0

**Published:** 2022-04-19

**Authors:** Mário Luciano de Mélo Silva Júnior, Marcelo Moraes Valença, Pedro Augusto Sampaio Rocha-Filho

**Affiliations:** 1 Division of Neuropsychiatry, Federal University of Pernambuco (UFPE), Recife, Brazil; 2 Neurology Unit, Hospital da Restauração, Recife, Brazil; 3 Medical School, Uninassau, Recife, Brazil; 4 Headache Clinic, Hospital Universitario Oswaldo Cruz, University of Pernambuco (UPE), Recife, Brazil

**Keywords:** Depression, Anxiety, Burnout, Medical residency, Learning, Diurnal somnolence, Moonlighting

## Abstract

**Background:**

Psychological distress is common among medical trainees. This study aimed to assess the frequency of depression, anxiety and burnout among physician residents and their association with both individual and residency program-related factors.

**Methods:**

This was a cross-sectional study applying an online survey in a national-wide sample of medical residents from Brazil. Depression, anxiety, burnout and diurnal somnolence were assessed with validated tools (Patient Health Questionnaire-4, 2 items version of Maslach Burnout Inventory, and Epworth Sleepiness Scale). Socio-demographic and residency program-related factors were measured with internally validated instruments. We performed multivariate binary logistic regression analysis for each of the main outcomes.

**Results:**

Screening for depression, anxiety and burnout was positive respectively in 46.9%, 56.6% and 37.0% of our sample (*n* = 1,419). Depression was independently related to female sex, longer duty hours, absence of day off, poor learning perception, poor feeling about the residency program, overall occurrence of psychological abuse, anxiety, diurnal somnolence and burnout (AUROC = .859 [95%CI = .840-.878], *p* < .001). Anxiety was independently associated with female sex, higher age and duty hours, work-personal life conflicts, few classroom activities, providing assistance without supervision, depression and diurnal somnolence (837 [.816-.857], *p* < .001). Burnout was related to lower age and leisure time, male sex, longer duty hours, absence of day off, provision of care without supervision, choice of the wrong specialty, poor learning, psychological abuse, depression and diurnal somnolence (.780 [.753-.806], *p* < .001).

**Conclusion:**

Frequency of psychological distress in residency training is high and related to both individuals and environmental factors, namely high workloads, occurrence of psychological abuse, poor faculty supervision, poor learning experience and work-personal life conflicts.

## Introduction

Medical residency is an important period in the qualification of medical specialists during which a physician is inserted into the workplace and creates bonds with employers and their peers. Although it is a period of acquiring knowledge, abilities, and competencies, there are heavy burdens of personal and external obligations, in tandem with the resident’s immaturity in handling responsibilities, and a complex moment in the life of the young, who are frequently subjected to personal demands, such as starting a family.

Medical residents have higher rates of suicidal ideation [1], migraine, sleep deprivation, anxiety, depression and work-personal life conflicts [2] than the general population. Those issues are related to negative impacts on patients’ care and safety [3]. Psychological distress in residency is a topic often explored in the literature, but there is a dearth of large, national, multiple-specialty studies on this subject.

Studies with residents have related the occurrence of anxiety, depression, and burnout mainly to individual characteristics, such as age, sex [4, 5] social skills [6], alcohol consumption [7] and genetic factors [8]. Furthermore, the research addressing the effect of residency program environment on psychological distress focuses mainly on the workoad [9–11]. Recently, some data have pointed out that faculty feedback and research ranking position [12] can decrease psychological distress, while mistreatment occurrence increases it [13]. The effect of other environmental aspects, such as attendings’ supervision, classroom activities and weekly day off – which are components of Brazilian standards for medical residency training – was not yet reported, so far we know.

This study set out to assess the frequency of anxiety, burnout and depression among Brazilian physician residents and their association with both individual and environmental factors, such as duty-hours, learning experience, attendings supervision, weekly day off, occurrence of mistreatment, and work-personal life conflicts.

## Methods

This was a national, cross-sectional, web-based survey, enrolling medical residents from 50 of the 55 medical specialties in Brazil. This was an exploratory study; a primary analysis and full description of the methods have been previously published [2].

Ethics Committee approval was obtained before data collection; all Brazilian ethical standards were followed. The only inclusion criterion was to be a medical resident at the time of completion of the survey; all volunteers gave their informed consent to participate in the study. No benefits were offered or given. The Strengthening the Reporting of Observational Studies in Epidemiology [14] and the Checklist for Reporting Results of Internet E-Surveys [15] reporting guidelines were followed.

We developed an online survey (on Google Forms) which was spread onto the pages of medical residents’ associations (Facebook and Instagram) under the nonspecific title “How is your medical residency program going?”, from November to December 2019. We applied the unique-individual click counter Bit.ly to assess the completion rate. No sample size calculation was performed before the data collection.

There were questions on socio-demographic and residency program-related aspects. These items were based on the Brazilian standards for medical residency programs and were internally validated applying qualitative methods (cognitive interviews using ﻿think-aloud and probing strategies) through both online and in-person meetings, with a pilot group of 20 medical residents (10 men and 10 women), from surgical, surgical clinics, medical and diagnostic areas, who were in post-graduate year (PGY) 1 to 4. Data obtained during this process were excluded from the analysis.

The final version of the survey had 46-items over 4 pages. All questions had to be answered, except for the items which might identify the individuals in question, leading to a low rate of missing data. It was an open survey and was tested and approved before fielding.

### Mistreatment

We asked “﻿Have you suffered or witnessed other residents suffering mistreatment during your residency program?”. We assessed three kinds of abuse: psychological, sexual and physical aggression. No definition of any of these mistreatments was offered.

Positive answers were further investigated on those who perpetrated the mistreatment (medical staff, patient, patient’s family, fellow residents, or administrative personnel). The frequency of the mistreatment was not assessed, due to limitations of the platform we used.

### Work-personal life conflicts

This item was assessed by the 5-point Likert-scale affirmation “﻿My routine in this medical residency program allows me enough time for my personal and family activities”. Responses ranged from “strongly disagree” to “strongly agree”. Residents who responded strongly disagree or disagree were considered as having work-personal life conflicts.

### Learning experience

“How do you evaluate the learning curve that you have experienced throughout this medical residency program?” was the question that assessed the learning perception of volunteers. Responses were obtained on a 5-item Likert scale ranging from “﻿very satisfactory” to “﻿very unsatisfactory”. Individuals who respond “very unsatisfactory” or “﻿unsatisfactory” were considered as having a poor learning experience.

### General feeling about the residency program

To evaluate this, we asked the question “In general, how do you feel about your residency program?” on a 5-item Likert scale ranging from “I am very dissatisfied” to “I am very satisfied”. Volunteers who responded “I am very dissatisfied” or “﻿I am dissatisfied” to this question were considered as having poor satisfaction with the residency program.

### Frequency of attendings’ supervision

“Have you provided medical assistance without attendings’ supervision on what frequency?” was a Likert-type question with replies ranging from “never” to “daily”. Volunteers who responded “most days in a week” or “daily” were considered as “often providing assistance without supervision”.

Regarding qualitative interviews, residents considered that the physical presence (not necessarily in the same room, but close) and availability of a qualified attending are needed to configure a supervised activity. This definition is similar to the ACGME for direct and indirect supervision [16].

### Frequency of classroom activities

We appraised this item by the question “On average, what is the frequency of classroom activities (seminars, critical appraisal of papers, others) in your residency program?”. Responses ranged from “I have not had any classroom activity in my residency program” to “More than four times per week”. Residents who responded “once a week” or less than that were labeled as having “few classroom activities”.

### Duty hours

Duty hours were assessed using the question “On average, what are the weekly duty hours of your residency program?”. Options ranged from “less than 60 h per week”, “About 60”, up to “more than 100 h per week”. Similarly, we also assessed the frequency of weekly duty hours on jobs unrelated to the residency requirements (moonlighting).

### Absence of a day off

We assessed it employing the question “In the past three months, have you had at least one day off per week?”. We offered four options ranging from “No, I have not had any days off” to “Yes, I have at least one day off every week”. Residents who responded “No, I have not had any days off” or “Yes, I have, but only in few weeks only” were considered as having “absence of days off”.

### Choosing the wrong specialty

Individuals who responded “yes, often” to the 3-item Likert-question “In the past three months, have you thought you chose the wrong medical specialty to pursue?” were labeled as “choosing the wrong specialty”.

### Leisure time

“Currently, on average, how many hours per week have you devoted to yourself (hobbies, physical exercise, beauty care, etc.)?”. Individuals marked a number ranging from 0 to 100 h.

### Patient Health Questionnaire-4 (PHQ-4)

This tool consists of four questions (two for depression, derived from PHQ-9 [Cronbach's alpha = 0.83] [17]; and two for anxiety, derived from GAD-9 [Cronbach's alpha = 0.81] [17]) which are scored from 0 to 3 for each question – a total score of up to 6 points for each domain. Individuals who scored 3 or more in each domain were screened positive, accordingly, for depression and anxiety. This cut-off has a pooled sensitivity of 89% and specificity of 76% for detecting depression [18]; and a sensitivity of 80% and specificity of 81% for detecting anxiety [19].

### Two items version of Maslach Burnout Inventory (MBI-2i)

The MBI-2i assesses two domains of burnout – depersonalization and emotional exhaustion – using a single question for each (single item to original MBI subscale correlation = 0.89 for each question [20]). Each question was scored on a 5-item Likert scale ranging from “No, I have not” to “Yes, every day”. Individuals who answered ﻿ “yes, every day” or “yes, some days a week” to both replies were considered to be burned out.

The emotional exhaustion domain was assessed by the question﻿ “Have you felt burned out in your residency program?” and depersonalization domain by the question “﻿Have you felt you became more callous toward people since you started this residency program?”, which both ﻿cover an area under the receiver operating curve of 94% and 95% [21], respectively, for each specific domain of the original MBI.

### Epworth Somnolence Scale (ESS)

We assessed daytime sleepiness through the 6-item Portuguese version of ESS (Cronbach's alpha = 0.83) [22]. Each question scores from 0 (no chance of dozing) to 3 (very likely to doze), so a total score from 0 to 18. Individuals who scored 10 or more were considered as having diurnal somnolence.

### Statistical analysis

We present data in three main dependent variables: depression, anxiety, and burnout groups. Individuals who were screened as not having any of these outcomes were considered controls for the respective outcome.

For ordinal and nominal variables data are reported in frequency and odds ratio (OR) and Fisher’s test was applied.

For discrete variables, median and interquartile range (IQR) was used, followed by Mann–Whitney’s test, as Kolmogorov–Smirnov’s test resulted in a non-parametric distribution of these variables. Variance inflation factor and matrix correlation were applied to assess multicollinearity.

A binary logistic multiple regression model was applied to assess the effect of independent variables (as dichotomized or discrete) on each screened outcome (depression, anxiety and burnout). All variables with *p* < 0.20 for each outcome in bivariate analysis were entered in the model. We performed a backward stepwise process for the exclusion of variables with the higher Wald p at each step until all factors were *p* < 0.05. We provide the area under the receiver operating curve (AUROC) values, adjusted odds ratios (OR_adj_) and its 95% confidence intervals (95%CI).

We used the SPSS v25 for MacOS for all analyses. ﻿The significance level of the statistical tests was set at *p* < 0.050 (two-tailed). This is an exploratory study and we did not prespecify an analysis plan, but we assumed the hypothesis that residency program environmental factors are related to psychological distress when developing the survey. Since our survey had to include virtually all the questions, our missing data was < 0.1%. Individuals with missing data were excluded from the specific analysis.

## Results

The link for our survey received ﻿1,989 clicks and 1,421 responses were registered (71.4% of all clicks), but two individuals were excluded due to conflicting responses, resulting in 1,419 valid answers. Residents from all geographic areas and 50 of the 55 (91%) medical specialties in Brazil responded to our survey. Table 1 shows the demographic data of our sample. No collinearity was identified among the dependent variables.

**Table 1:** Demographic data

Variable	Overall (*n* = 1,419)
Age (years-old, median, IQR)	28 (27–30)
Female sex^a^ (n, %)	866 (61.0)
Sexual orientation, heterosexual^b^ (n, %)	1256 (88.5)
Single (n, %)	978 (68.9)
Have children (n, %)	127 (9.0)
Live with (n, %)	
Alone	540 (38.1)
Partner/children	458 (32.3)
Parents	304 (21.4)
Friends	117 (8.3)
Moving to participate in this residency (n, %)	913 (64.3)
Geographic region of residency program (n, %)	
South/Southeast	972 (68.5)
North/Northeast/Midwest	447 (31.5)
Training area (n, %)	
Diagnostic	73 (5.2)
Surgical	610 (43.0)
Clinical	736 (51.9)
Post-graduation year (median, IQR)	2 (1–3)
First year	571 (40.2)
Second year	422 (29.7)
Third year	286 (20.2)
Fourth year or more	140 (9.9)

^a^ 18 and ^b^ 23 volunteers did not reply to these questions

### Depression

Symptoms of depression occurred in 666/1,419 (46.9%) of our sample. Table 2 shows demographic, residency program-related aspects, as well as the occurrence of psychological and sexual abuses and physical aggression associated with depression.

**Table 2:** Associations between characteristics of residents, residency program-related factors, occurrence of abuses and presence of depression

**Variable**	**Depression (*n* = 666)**	**Controls (*n* = 753)**	**OR (95%CI)**	**p**^a^	**OR**_**adj**_** (95%CI)**^b^	**p**^b^
Age (years, median, IQR)	28 (27–30)	28 (27–30)	-	.823	-	
Female sex (n, %)	449 (67.4)	417 (55.4)	1.78 (1.43–2.21)	< .001	1.69 (1.27–2.26)	< .001
Non-heterosexual (n, %)	75 (11.4)	65 (8.8)	1.34 (.94–1.90)	.109	-	
Marital status single (n, %)	468 (70.3)	510 (67.7)	1.13 (.90–1.41)	.329	-	
Have children (n, %)	50 (7.5)	77 (10.2)	.71 (.49–1.03)	.077	-	
Live alone (n, %)	265 (39.8)	275 (36.5)	1.15 (.93–1.42)	.208	-	
Move to participate in this residency (n, %)	430 (64.6)	483 (64.1)	1.02 (.82–1.27)	.912	-	
Surgical training area (n, %)	293 (44.0)	317 (42.1)	1.08 (.88–1.33)	.485	-	
Post-graduation year (median, IQR)	2 (1–3)	2 (1–3)	-	.653	-	
Weekly working hours in residency (median, IQR)	70 (60–80)	60 (60–80)	-	< .001	1.09 (1.00–1.20)	.049
Weekly moonlighting hours (median, IQR)	9 (3–18)	9 (3–18)	-	.816	-	
Leisure time (hours/week, median, IQR)	5 (2–8)	6 (4–12)	-	< .001	-	
Absence of day off (n, %)	283 (42.5)	207 (27.5)	1.95 (1.56–2.43)	< .001	1.41 (1.04–1.90)	.027
Poor learning experience (n, %)	293 (44.0)	122 (16.2)	4.06 (3.17–5.20)	< .001	1.87 (1.32–2.66)	< .001
Work-personal life conflicts (n, %)	563 (84.5)	540 (71.7)	2.16 (1.66–2.81)	< .001	-	
General poor feeling about residency program (n, %)	233 (35.0)	63 (8.4)	5.89 (4.35–7.89)	< .001	2.40 (1.58–3.64)	< .001
Few classroom activities (n, %)	377 (57.2)	349 (46.7)	1.53 (1.24–1.89)	< .001	-	
Providing assistance without supervision often (n, %)	451 (67.7)	332 (44.1)	2.66 (2.14–3.31)	< .001	-	
Choosing the wrong specialty (n, %)	363 (54.5)	149 (19.8)	4.86 (3.84–6.15)	< .001	-	
Occurrence of psychological abuse (n, %)	505 (75.8)	383 (50.9)	3.03 (2.41–3.81)	< .001	1.64 (1.23–2.20)	.001
Occurrence of psychological abuse from attendings (n, %)	361 (54.2)	225 (29.9)	2.78 (2.23–3.46)	< .001	-	
Occurrence of psychological abuse from patients (n, %)	161 (24.2)	132 (17.5)	1.50 (1.16–1.94)	.002	-	
Occurrence of psychological abuse from colleague residents (n, %)	156 (23.4)	88 (11.7)	2.31 (1.74–3.08)	< .001	-	
Occurrence of sexual abuse in your residency program (n, %)	221 (33.2)	197 (26.2)	1.40 (1.12–1.76)	.004	-	
Occurrence of sexual abuse in your residency program from attendings (n, %)	101 (15.2)	56 (7.4)	2.23 (1.58–3.14)	< .001	-	
Occurrence of physical aggression in your residency program (n, %)	130 (19.5)	108 (14.3)	1.45 (1.10–1.92)	.010	-	
Anxiety (n, %)	569 (85.4)	234 (31.1)	13.0 (9.98–17.0)	< .001	8.61 (6.45–11.48)	< .001
Diurnal somnolence† (n, %)	499 (74.9)	397 (52.7)	2.68 (2.14–3.36)	< .001	1.46 (1.09–1.97)	.012
Burnout (n, %)	359 (53.9)	166 (22.1)	4.14 (3.29–5.20)	< .001	2.59 (1.93–3.49)	< .001

^a^ Fisher exact or Mann–Whitney tests

^b^ Final binary logistic regression model: AUROC = .859 (95%CI = .840-.878)

This model enrolled 1,398 individuals (21 [1,5%] had at least one missing variable)

Individuals with depression were more frequently women, had longer weekly duty hours (median of 10 h longer), poorer general feeling about training and subjective learning curve, as well as reported psychological abuse and absence of a weekly day off more frequently. Anxiety, burnout, and diurnal somnolence were also associated with depression.

### Anxiety

Anxiety occurred in 803/1,419 (56.6%) of our respondents. Table 3 depicts demographic, psychological distress aspects and residency program-related variables according to anxiety status.

**Table 3:** Associations between characteristics of residents, residency program-related factors, occurrence of abuses and presence of anxiety

**Variable**	**Anxiety** **(*n* = 803)**	**Controls (*n* = 616)**	**OR (95%CI)**	**p**^a^	**OR**_**adj**_** (95%CI)**^b^	**p**^b^
Age (years, median, IQR)	28 (27–31)	28 (26–30)	-	.070	1.06 (1.02–1.11)	.004
Female sex (n, %)	526 (66.8)	340 (55.4)	1.62 (1.31–2.02)	< .001	1.37 (1.04–1.80)	.025
Non-heterosexual (n, %)	79 (10.0)	61 (10.1)	1.01 (.71–1.43)	.99	-	
Marital status single (n, %)	558 (69.5)	420 (68.2)	1.06 (.85–1.33)	.603	-	
Have children (n, %)	78 (9.7)	49 (8.0)	1.25 (.86–1.81)	.262	-	
Live alone (n, %)	316 (39.4)	224 (36.4)	1.14 (.91–1.41)	.270	-	
Move to participate in this residency (n, %)	520 (64.8)	393 (63.8)	1.04 (.84–1.30)	.737	-	
Surgical training area (n, %)	367 (45.7)	243 (39.4)	1.29 (1.04–1.60)	.020	-	
Post-graduation year (median, IQR)	2 (1–3)	2 (1–3)	-	.838	-	
Weekly working hours in residency (median, IQR)	70 (60–80)	60 (60–70)	-	< .001	1.15 (1.05–1.27)	.004
Weekly moonlighting hours (median, IQR)	9 (3–18)	9 (3–18)	-	.791	-	
Leisure time (hours/week, median, IQR)	5 (2–10)	7 (4–12)	-	< .001	-	
Absence of day off (n, %)	322 (40.1)	168 (27.3)	1.79 (1.42–2.24)	< .001	-	
Poor learning experience (n, %)	307 (38.2)	108 (17.5)	2.91 (2.26–3.75)	< .001	-	
Work-personal life conflicts (n, %)	681 (84.8)	422 (68.5)	2.57 (1.99–3.32)	< .001	1.57 (1.13–2.17)	.007
General poor feeling about residency program (n, %)	237 (29.5)	59 (9.6)	3.95 (2.91–5.38)		-	
Few classroom activities (n, %)	455 (57.1)	271 (44.4)	1.66 (1.35–2.06)	< .001	1.30 (1.00–1.70)	.049
Providing assistance without supervision often (n, %)	523 (65.1)	260 (42.2)	2.56 (2.06–3.12)	< .001	1.57 (1.20–2.05)	.001
Choosing the wrong specialty (n, %)	374 (46.6)	138 (22.4)	3.02 (2.39–3.82)	< .001	-	
Occurrence of psychological abuse (n, %)	581 (72.4)	307 (49.8)	2.63 (2.11–3.29)	< .001	-	
Occurrence of psychological abuse from attendings (n, %)	406 (50.6)	180 (29.2)	2.48 (1.98–3.09)	< .001	-	
Occurrence of psychological abuse from patients (n, %)	190 (23.7)	103 (16.7)	1.54 (1.18–1.02)	.001	-	
Occurrence of psychological abuse from colleague residents (n, %)	172 (21.4)	72 (11.7)	2.06 (1.53–2.78)	< .001	-	
Occurrence of sexual abuse in your residency program (n, %)	263 (32.8)	155 (25.2)	1.45 (1.15–1.83)	.002	-	
Occurrence of sexual abuse in your residency program from attendings (n, %)	105 (13.1)	52 (8.4)	1.63 (1.15–2.32)	.006	-	
Occurrence of physical aggression in your residency program (n, %)	154 (19.2)	84 (13.6)	1.50 (1.13–2.01)	.006	-	
Depression (n, %)	569 (70.9)	97 (15.7)	13.01 (9.98–17.0)	< .001	10.17 (7.70–13.14)	< .001
Diurnal somnolence† (n, %)	596 (74.2)	300 (48.7)	3.03 (2.43–3.79)	< .001	1.84 (1.39–2.44)	< .001
Burnout (n, %)	388 (48.3)	137 (22.2)	3.27 (2.58–4.14)	< .001	-	

^a^ Fisher exact or Mann–Whitney tests

^b^ Final binary logistic regression model: AUROC = .837 (95%CI = .816-.857)

This model enrolled 1,386 individuals (33 [2,3%] had at least one missing variable)

Individuals with anxiety were also more frequently women, and older, had more frequent diurnal somnolence, unsatisfactory work-personal life balance and depression. Operational aspects of the residency program, such as few classroom activities, longer duty hours and assisting frequently without supervision were also independently related to anxiety.

### Burnout

Burnout occurred in 525/1,419 (37.0%) of our sample; the emotional domain was present in 995/1,419 (70.1%), and depersonalization in 534/1,419 (37.6%).

Table 4 shows a detailed analysis of burnout in medical residents. Individuals with burnout were younger, more frequently men, and had less leisure time, in tandem with longer duty hours and more diurnal somnolence. Environmental factors such as the absence of a day off, provision of assistance without supervision and psychological abuse from patients and attendings were more frequent in the burnout group than in controls. Volunteers with burnout presented higher odds of depression and of reported that they had a poor learning curve as well as had chosen the wrong specialty.

**Table 4:** Associations between characteristics of residents, residency program-related factors, occurrence of abuses and presence of burnout

**Variable**	**Burnout (*n* = 525)**	**Controls (*n* = 894)**	**OR (95%CI)**	**p**^a^	**OR**_**adj**_** (95%CI)**^b^	**p**^b^
Age (years, median, IQR)	28 (26–30)	28 (27–30)	-	.083	.94 (.90–99)	.010
Male sex (n, %)	226 (43.6)	309 (35.0)	1.44 (1.15–1.80)	.001	2.11 (1.58–2.81)	< .001
Non-heterosexual (n, %)	50 (9.7)	90 (10.2)	1.06 (.74–1.53)	.782	-	
Marital status single (n, %)	376 (71.6)	602 (67.3)	1.22 (.97–1.55)	.096	-	
Have children (n, %)	38 (7.2)	89 (10.0)	.71 (.48–1.05)	.101	-	
Live alone (n, %)	210 (40.0)	330 (36.9)	1.14 (.91–1.42)	.314	-	
Move to participate in this residency (n, %)	346 (65.9)	567 (63.4)	1.12 (.89–1.40)	.359	-	
Surgical training area (n, %)	266 (50.7)	344 (38.5)	1.64 (1.32–2.04)	< .001	-	
Post-graduation year (median, IQR)	2 (1–3)	2 (1–3)		.925	-	
Weekly working hours in residency (median, IQR)	70 (60–90)	60 (60–80)	-	< .001	1.20 (1.10–1.32)	< .001
Weekly moonlighting hours (median, IQR)	9 (3–18)	9 (3–18)	-	.460	-	
Leisure time (hours/week, median, IQR)	4 (2–8)	6 (4–12)	-	< .001	.98 (.96–1.00)	.044
Absence of day off (n, %)	242 (46.1)	248 (27.7)	2.23 (1.78–2.79)	< .001	1.34 (1.00–1.79)	.047
Poor learning experience (n, %)	223 (42.5)	192 (21.5)	2.70 (2.13–3.42)	< .001	1.48 (1.09–2.02)	.013
Work-personal life conflicts (n, %)	455 (86.7)	648 (72.5)	2.47 (1.84–3.30)	< .001	-	
General poor feeling about residency program (n, %)	168 (32.0)	128 (14.3)	2.82 (2.17–3.66)	< .001	-	
Few classroom activities (n, %)	302 (58.0)	424 (47.9)	1.50 (1.21–1.87)	< .001	-	
Providing assistance without supervision often (n, %)	365 (69.5)	418 (46.8)	2.60 (2.07–3.26)	< .001	1.32 (1.00–1.76)	.049
Choosing the wrong specialty (n, %)	280 (53.3)	232 (26.0)	3.26 (2.60–4.09)	< .001	1.77 (1.32–2.35)	< .001
Occurrence of psychological abuse (n, %)	410 (78.1)	478 (53.5)	3.10 (2.43–3.96)	< .001	-	
Occurrence of psychological abuse from attendings (n, %)	303 (57.7)	283 (31.7)	2.95 (2.36–3.68)	< .001	1.56 (1.21–2.13)	.002
Occurrence of psychological abuse from patients (n, %)	142 (27.0)	151 (16.9)	1.82 (1.41–2.37)	< .001	1.57 (1.13–2.17)	.006
Occurrence of psychological abuse from colleague residents (n, %)	142 (27.0)	102 (11.4)	2.88 (2.17–3.82)	< .001	-	
Occurrence of sexual abuse in your residency program (n, %)	198 (37.7)	220 (24.6)	1.86 (1.47–2.34)	< .001	-	
Occurrence of sexual abuse in your residency program from attendings (n, %)	91 (17.3)	66 (7.4)	2.63 (1.88–3.69)	< .001	-	
Occurrence of physical aggression in your residency program (n, %)	119 (22.7)	119 (13.3)	1.91 (1.44–2.53)	< .001	-	
Anxiety (n, %)	388 (73.9)	415 (46.4)	3.27 (2.58–4.14)	< .001	-	
Depression (n, %)	359 (68.4)	307 (34.3)	4.14 (3.29–5.20)	< .001	2.53 (1.89–3.38)	< .001
Diurnal somnolence† (n, %)	397 (75.6)	499 (55.8)	2.46 (1.93–3.12)	< .001	1.52 (1.13–2.04)	.006

^a^ Fisher exact or Mann–Whitney tests

^b^ Final binary logistic regression model: AUROC = .780 (95%CI = .753-.806)

This model enrolled 1,398 individuals (21 [1,5%] had at least one missing variable)

We did not observe any association between burnout (nor for depression and anxiety) and PGY or any specific training area.

### Co-occurrence of depression, anxiety and burnout

Table 5 depicts the Spearman correlation coefficients for interaction among depression, anxiety and burnout (as well as burnout domains). Observe that due to the criterion applied to define burnout, depersonalization was highly correlated to burnout.

**Table 5:** Spearman correlation (r) among depression, anxiety, burnout (and emotional exhaustion and depersonalization domains) and diurnal somnolence screenings

Variable	Depression	Anxiety	Burnout	Burnout domain	
				**Emotional**	**Depersonalization**
Anxiety	.547	-			
Burnout	.329	.267	-		
Emotional domain	.283	.316	.235	-	
Depersonalization domain	.318	.254	.987	.214	-
Diurnal somnolence	.230	.262	.198	.263	.186

All of the interactions presented a p-value < .001.

The frequency of co-existing depression and anxiety was 569/1,419 (40.1%). Depression and burnout co-occurred in 359/1,419 (25.3%) individuals. Both anxiety and burnout were present in 388/1,419 (27.3%) volunteers.

## Discussion

To the best of our knowledge, this is the largest study assessing psychological distress in medical residents in South America, enrolling volunteers from all geographic areas of Brazil and almost all medical specialties. Our data, in line with the literature, showed high frequencies of depression, anxiety and burnout: 46.9%, 56.6% and 37.0%, respectively. Our data reinforce the burden of mental illness in this population and point to some residency program factors that may be addressed to reduce the risk of psychological distress and improve the general satisfaction of residents.

Meta-analysis data showed a pooled prevalence of depression ranging from 20.8% to 43.2% and a significant increase rate was observed over time [23, 24].

The prevalence of anxiety in residents ranges from 11 to 74% [4] depending on the tools applied, areas of training and countries studied.

Pooled prevalence of burnout in medical residents ranges from 35.7% to 51.0% [5], which is in line with our findings. Burnout construct is complex and different definitions and settings promote this range.

Rates of depression increase substantially during residency training [12]. Individual, but mainly extrinsic factors are related to this. Female sex is often highlighted as a risk factor for depression in residents in terms of frequency and severity [24]; however, age, medical specialty and post-graduate year do not seem to influence the occurrence of depression [23].

According to our data and a large Japanese study, there is a quasi-linear relation between increasing duty hours and depression [9], confirmed on prospective data [12]. Depression and overwork also occur in other populations [25] owing to a complex interaction of imbalance among high job demands and poor rewards from work, less leisure time, more work-family conflicts, and poor sleep. In fact, diurnal somnolence and the absence of at least one day off were independently related to depression in our data.

Sleep quality decreases along with residency training due to on-call shifts and studying cases. There are data on the negative impact of sleep deprivation on cognitive function and learning in different settings [26], as well as its correlates to depression and anxiety [7].

Mistreatment in the workplace is a risk factor for depression [27]. Among residents, public belittlement and humiliation are common forms of mistreatment. We evaluated the concept of being mistreated or witnessing mistreatment, which points more to the work environment than to individual relationships.

However, it should be mentioned that sleepiness, poor self-perception of learning and noticing more mistreatment could be a symptom of depression.

As we observed, a higher frequency of anxiety among women is often reported [4], but its association with training areas and post-graduate year are not consistent [7].

The association between long working hours and anxiety also occurs in the general population [28]. There is an association between resident duty hours, the number of patients being cared for, and insufficient supervision [29]. We hypothesize that foreseeing problems while medically assisting without faculty supervision may predispose to an anxiety state. High workloads may also lead to stress and sleep disturbances [30], contributing to anxiety. Other studies found a rise in anxiety with increasing age [4] probably due to interactions with extra-residency factors, such as work-personal life conflicts.

Studies addressing the role of supervision by attendings or classroom activities addressing anxiety are lacking. We hypothesize that such activities may consolidate learning and engage the team which could mitigate anxiety.

Different definitions of burnout and selection criteria have led to different results on recent meta-analyses of sex, age, post-graduation year, and medical specialty as risk factors [5, 31]. The effect of a post-graduation year is probably more influenced by duty hours than by PGY itself.

Men seem to be more affected by workloads and the depersonalization domain [11], while women are more likely to experience work-personal life conflicts and the emotional exhaustion domain [10, 13]. Age as a protection factor may reflect those conflicts since our sample is composed chiefly of single individuals. Our findings corroborate a recent review [31] indicating that there is no specific group of specialties leading to burnout – or depression and anxiety – but there are different patterns of burnout domains in each specific specialty.

We observed that burnout was related to longer working hours, absence of days off, less leisure time and diurnal somnolence, aspects relating to excessive work requests which are associated with a feeling of lack of professional accomplishment, as the idea of having chosen the wrong specialty. Another factor related to burnout is extensive interpersonal conflicts at work, which agrees with our findings of psychological abuse from attendings and patients, also observed by others [13]. The relationship between inadequate supervision and increased burnout has already been reported [10] and may represent a poor learning environment and resident overwork.

Burned-out physicians engage less in their activities and with patients [11], which may be related to an insufficient learning experience during training, as we observed. The effect of higher levels of burnout was already linked to lower scores in performance on objective evaluation, an impairment comparable to the knowledge expected to be acquired over an entire year [32].

In literature there are correlates among depression and burnout and anxiety [6, 7] in line with ours, indicating a state of mental distress during residency more than a specific diagnosis.

Psychological distress is correlated with several factors, intrinsic and environmental. These exposures and results we found could be similar in other populations, mostly those similar in workloads and stress levels, such as nurses, police officers, and firefighters.

Residency program aspects could be addressed by policymakers, but also program coordinators and faculty members, to enhance the training environment. It is possible to schedule classroom activities, conferences and feedback sessions, improve attending's supervision with andragogical learning techniques, and tailor activities to engage the team. There is a need to achieve a balance between skills acquirements, residents’ wellbeing and quality of patient care. Regulatory institutions could also be aware that duty-hours violations are related not only to physical stress but psychological issues. So, they should reinforce the ways to guarantee that the limits be respected.

Individual factors may be focused on resilience strengthening and cognitive processing. The stigma of mental illness should be clarified, and professional assistance could be offered to all residents. Our results may help clinicians’ approach to those individuals, once we highlighted some major elements that play roles in this process.

Our study has a number of limitations. It was a convenience sample, and a selection bias cannot be excluded. Despite this, our sample is comparable to Brazilian census data [33] on distribution of age, sex, geographical region and training areas and is equivalent to 4% of Brazilian residents. The social media spread of our survey could lead to non-residents responding to it and a *priori* not all residents are potentially accessible through this system. We applied short versions of screening tools, which tends to diminish accuracy and it is not diagnostic of any of the outcomes measured. Questions related to residency programs were not externally validated and could be addressed by further studies. View, recruitment and completion rates could not be measured.

## Conclusion

Depression, anxiety, and burnout in medical residents are frequent and related to individual factors such as age and sex, but also to residency programs aspects, such as long duty hours, the occurrence of mistreatment, poor learning experience, provision of assistance without proper supervision, work-personal life conflicts, few classroom activities, and absence of at least one day off per week.

## Data Availability

The datasets used and analyzed during the current study are available from the corresponding author on reasonable request.

## Abbreviations

PHQ-4: Patient Health Questionnaire-4
MBI-2i: Two items version Maslach Burnout Inventory
ESS: Epworth Somnolence Scale
PGY: Post-graduation year
